# Effects of Different Levels of Carrot Juice Supplementation in Drinking Water on Performance, Egg Quality, Blood Parameters, and Gut Health of Babcock White Laying Hens

**DOI:** 10.3390/life16030382

**Published:** 2026-02-27

**Authors:** Umit Ozcinar, Muhammet Emre Orman, Cangir Uyarlar, Eyup Eren Gultepe, Oğuz Kağan Türedi, Aamir Iqbal, İbrahim Sadi Çetingül, Beytullah Kenar, Ismail Bayram

**Affiliations:** 1 Department of Animal Nutrition and Nutritional Diseases, Faculty of Veterinary Medicine, Afyon Kocatepe University, Afyonkarahisar 03200, Türkiye; umitozcinar@gmail.com (U.O.); cangiruyarlar@hotmail.com (C.U.); aamir_vet@yahoo.com (A.I.); sadicet@yahoo.com (İ.S.Ç.); ibayram1965@gmail.com (I.B.); 2 Kemin Europa NV, Toekomstlaan 42, 2200 Herentals, Belgium; eegultepe@gmail.com; 3 Department of Microbiology, Faculty of Veterinary Medicine, Afyon Kocatepe University, Afyonkarahisar 03200, Türkiye; oguz_kaan_turedi@hotmail.com (O.K.T.); beytullahkenar@yahoo.co.uk (B.K.)

**Keywords:** carrot juice, drinking water, egg quality, gut microbiota, laying hens

## Abstract

In this study, the effects of carrot juice on performance, egg quality, blood parameters, and intestinal flora in laying hens were examined. One hundred twenty-eight Babcock white laying hens were divided into four random groups, each with four replicates of eight hens. Fresh carrot juice was introduced to the drinking water of the hens at concentrations of 0% (control), 1%, 2.5%, and 5% for a duration of four weeks. Weekly feed intake, egg weight, feed conversion ratio, and daily egg production were monitored, while yolk color, albumen and yolk indices, shell thickness, and Haugh units were measured in weeks two and four. Blood samples were analyzed for biochemical and physiological parameters, and fecal samples were analyzed for microbial parameters at the end of the study. Carrot juice improved egg production, egg weight, and egg mass, without affecting daily feed intake, feed conversion ratio, or water consumption, while enhancing yolk color but reducing eggshell thickness. Blood analyses showed higher levels of total oxidant status, vitamin E, immunoglobulin G, alkaline phosphatase, lymphocyte count, and mean corpuscular hemoglobin concentration. Moreover, fecal Enterococcus counts were positively affected by the treatment. The findings indicate that carrot juice supplementation could positively affect performance, egg quality, some immune-related blood parameters, and gut microbiota in laying hens. Among the levels evaluated, adding 2.5% carrot juice to drinking water was found to provide the most balanced response.

## 1. Introduction

Poultry production has faced challenges from many factors, including food safety, environmental issues, welfare standards, ban of non-therapeutic in-feed antibiotics, gut health, feeding high-fiber-containing ingredients, and maintaining high efficiency of production in recent years [1]. This situation has led to studies on the development of management and nutrition strategies that improve the productivity and quality of the product without any impairment in the health status of animals [2]. The use of feed additives able to improve the efficiency of growth and/or egg production, prevent disease, and enhance feed utilization is an option for handling the aforementioned challenges [1]. However, nowadays, the use of plant-based natural feed additives has gained importance because of some concerns regarding the impact of traditional feed additives on public health and safety [3]. The supplementation of plant-based natural additives into poultry diets offers some advantages due to their growth-promoting, anti-microbial, anti-inflammatory, immunostimulant, and egg-production-promoting properties, as well as their nutrient composition [4,5].

Carrot is a vegetable that possesses nutraceutical effects and some health benefits due to its natural bioactive substance composition. While the bioactive substances of carrot include flavonoids, phenolics, ascorbic acid, and polyacetylene, its high β-carotene content is the main contributor to its nutritional benefits [6]. Steenfeldt et al. [7] reported that carrot silage addition to the rations of laying hens increased egg production and reduced mortality rates. Sarmiento-Garcia et al. [8] found that carrot supplementation improved eggshell weight, eggshell thickness, and antioxidant status in quails. Additionally, the use of carrot meal in broiler diets offered some advantages, including improved feed intake, feed conversion ratio, metabolizable energy intake, and nitrogen retention [9].

One of the available forms of carrot to use in animal feeds is carrot juice because of its similarity to roots in terms of nutrient content. Carrot juice supplementation via drinking water is a beneficial supplement in broilers fed a diet containing clove meal because of its antioxidant content [10]. It was shown that carrot juice improved antioxidant status and could be used to prevent some illnesses [11,12]. Many studies have evaluated the effects of various carrot products, such as meal, powder, or silage. However, to the best of our knowledge, studies evaluating the effects of carrot juice on the health and production parameters of laying hens are quite limited. Therefore, the objective of this study was to examine the effects of carrot juice addition in the drinking water of laying hens on performance, egg quality, blood parameters, and intestinal microflora.

## 2. Materials and Methods

This study was conducted in Afyonkarahisar, Türkiye (38.7011° N, 30.5233° E). Research on animals was conducted according to the Animal Experiments Local Ethics Committee of Afyon Kocatepe University (protocol number: AKUHADYEK-117-16 and AKUHADYEK-158-17, dated 20 October 2016 and 16 February 2017, respectively).

### 2.1. Birds and Experimental Design

A total of 128 fifty-week-old Babcock white laying hens were used in this study. The hens were randomly divided into four groups (3 treatments and 1 control). Each group was further divided into 4 subgroups consisting of 8 hens each. The hens were fed the same basal ration prepared in accordance with the NRC [13] recommendations (Table 1). The hens were housed in battery cages in a temperature- and humidity-controlled experimental hen house. The cage dimensions were 48 cm (width) × 45 cm (depth) × 45 cm (height), with two hens per cage, providing a stocking density of 1080 cm^2^ per hen. The carrot juice supplementation was initiated at the beginning of the experimental period, which lasted for four weeks. Feed and water were both provided ad libitum. A light–dark cycle consisting of 16 h of light and 8 h of darkness was adopted.

Fresh carrots were thoroughly washed with potable water to remove visible contaminants before juice extraction. No chemical sanitizing agents were used to avoid any chemical residue. After this, the juice was filtered to exclude any residue. The extraction process was performed every day during the study period, and drinking water was prepared fresh every day. While preparing the drinking water, the refuse from the previous day was removed by draining, and the water tank was refilled. Then, the carrot juice was supplemented at the ratios of 0% (control), 1%, 2.5%, and 5% of drinking water. A nipple drinking system attached to separate tanks was utilized to provide drinking water for each group. The chemical composition of the carrot juice was analyzed by HPLC using the method modified by Akbalık et al. [14]. The chemical constituents of the carrot juice are presented in Table 1.

### 2.2. Sampling, Data Collection, and Analysis

Performance parameters were monitored weekly, whereas egg quality traits were evaluated at wk 2 and wk 4 to capture both early and cumulative effects of the treatment. Additionally, the weights of all hens were recorded at both the beginning and end of the study. All collected eggs were recorded daily. The performance parameters were evaluated as indicated by Mahfuz et al. [15]. Hen–day egg production (%) calculations were made by dividing the total number of eggs produced by the number of hens and expressed as a percentage. Egg weight was recorded every week on a per-hen basis throughout the experimental period. Egg mass was found by multiplying egg weight by hen–day egg production (%). Feed intake was recorded weekly as the difference between the amount of feed provided and the feed remaining in the feeder units. To calculate the feed conversion ratio (FCR), feed intake was divided by egg mass.

At the end of the second and fourth weeks of the experimental period, three eggs per subgroup were collected and transported to the laboratory to determine their yolk color index, albumen index, yolk index, eggshell thickness, and Haugh unit values. To calculate the Haugh unit, albumen height was used (Digital Caliper, CD-15CP, Mitutoyo Ltd., Hampshire, UK) as described by Haugh [16]. Egg yolk color was evaluated by utilizing the Improved Roche Yolk Color Fan (Yolk-Fan™, DSM Nutritional Products AG, Kaiseraugst, Switzerland), which uses a 15-band color scale to evaluate yolk color. Albumen and yolk index values were calculated according to the methods reported by Tilki and Saatci [17] using the following formulae:

Albumen index = Albumen height (mm)/[Albumen length (mm) + Albumen width (mm)] × 100

Yolk index = Yolk height (mm)/Yolk diameter (mm) × 100.

Blood biochemical and physiological parameters were analyzed at the end of the study to understand the long-term physiological adaptations. At the time of completing the study, three hens per subgroup were selected for blood sampling. Samples were collected from the heart directly to measure physiological and biochemical parameters. Physiological parameters were analyzed using a compact hematology analyzer (BC 2800 Vet, Mindray Medical International Ltd., Shenzen, China). The physiological parameters measured in this study were lymphocyte counts (LCs), neutrophil counts (NCs), total leukocyte counts (TLCs), red blood cell counts (RBCs), monocyte counts (MCs), mean corpuscular volume (MCV), hemoglobin concentration (He), mean corpuscular hemoglobin concentration (MCHC), platelets (PLTs), hematocrit (HC), and mean platelet volume (MPV). The supernatant obtained by centrifuging the samples was used for biochemical analyses. An ELISA analyzer (Elisys Uno, Human mbH, Wiesbaden, Germany) was used to measure high-density lipoprotein (HDL), low-density lipoprotein (LDL), glucose, total protein (TPRO), total cholesterol (CHO), alanine aminotransferase (ALT), aspartate aminotransferase (AST), alkaline phosphatase (ALP), phosphorus, calcium, vitamin E, total oxidant status (TOS), total antioxidant status (TAS), and immunoglobulin G (IgG).

### 2.3. Microbiological Analysis

Microbiological evaluation was performed mid-experiment and at the end of the experimental period. Fecal samples were collected from four hens per subgroup on days 15 and 30 of the trial. Samples were randomly and individually obtained using sterile fecal containers and transported under cold chain conditions to the Microbiology Laboratory of the Veterinary Diagnosis and Analysis Center, Afyon Kocatepe University, for microbiological analyses. Subsequently, 1 g of each fecal sample was diluted in sterile saline at a ratio of 1:10 using a total of 12 Falcon tubes. The agars for microbiological culturing were formulated based on standardized conditions to examine total aerobic bacteria, *Lactobacillus* spp., *Enterococcus* spp., and the existence of *Salmonella* spp. and *Clostridium perfringens*. The appropriate dilution rates for each bacterial sample were determined by a pilot study based on their multiplication rates to ensure accurate results. The dilutions were 10^−8^ and 10^−10^ for PCA (total aerobic plate count), 10^−6^ and 10^−8^ for MRS (*Lactobacillus* spp.), 10^−6^ and 10^−8^ for SBM (*Enterococcus* spp.), 10^−6^ and 10^−8^ for PBLS (*Salmonella* spp.), and 10^−6^ and 10^−8^ for *Clostridium perfringens*, according to the methodology explained by Siriken et al. [18]. The samples were incubated at species-specific optimal temperatures under anaerobic or aerobic conditions. Subsequently, bacterial colony counts were performed, and the data were transferred to a digital environment for further analysis. Detailed information regarding the microbiological analysis procedures is given in Table 2.

### 2.4. Statistical Analysis

Egg production, body weight, egg weight, egg mass, feed intake, water consumption, feed conversion ratio (FCR), and egg quality parameters were analyzed using the JMP statistical analysis program (JMP, 2003, Version 5.0). These variables were evaluated using a two-way repeated measures analysis of variance (ANOVA), including the fixed effects of treatment, time, and their interaction. Multiple comparisons were performed using Tukey’s post hoc test. Blood biochemical and physiological parameters, which were measured at a single time point, were subjected to analysis of variance (ANOVA), followed by Student’s t-test for pairwise comparisons, employing the same statistical program.

Fecal microbiological data were analyzed using MedCalc software (version 18; MedCalc Software bvba, Ostend, Belgium). The effects of treatment and time were evaluated using two-way ANOVA and the Tukey–Kramer multiple comparison test.

The results are presented as values of mean ± standard error of the mean (SEM), and the statistical significance level was set at *p* < 0.05.

## 3. Results

Mean daily egg production and egg mass values increased in all carrot juice-supplemented groups (*p* < 0.0001 and *p* = 0.0037, respectively). Nevertheless, there were no statistically significant differences among the supplementation doses. Egg weight increased only in the 1% supplementation group, while the same effect was not observed at increasing doses of carrot juice (*p* = 0.0452). The live weights of the hens did not change significantly up to 5% supplementation, but the live weights measured in the 5% group were significantly lower than those measured in the other groups (*p* = 0.0411). Mean daily feed intake, FCR, and water consumption values were not significantly influenced by the treatments (Table 3). No significant time effect was observed on the performance parameters, except for the live weights of the hens. The effect of the interaction between time and treatment was not statistically significant (Table 3).

While the Haugh unit was greater in the 2.5% supplementation group compared to the other groups (*p* = 0.0089), egg yolk color values were higher in the 1% supplementation group (*p* = 0.0001). For these two parameters, there were no statistically significant differences between the control and the other treatment groups (Table 3). Eggshell thickness values were significantly smaller in the 1% group in comparison to the others, while they were similar to each other in the other treatment groups and the control group (*p* = 0.0213). The albumen index values in the 5% supplementation group were smaller compared to those in the 2.5% supplementation group. On the other hand, these values in the 5% and 2.5% groups did not significantly differ from the ones in the control and 1% groups. The treatments did not have a significant effect on the egg yolk index values (Table 3). A time effect was observed in the Haugh unit, egg yolk color, and albumen index, whereas mean eggshell index and egg yolk index values did not change significantly depending on time (*p* > 0.05). The effect of the time–treatment interaction was significant for all parameters except for eggshell thickness.

The serum biochemical parameter measurements are presented in Table 4. The TOS values of all treatment groups were higher in comparison to those in the control group (*p* = 0.0151). On the other hand, the TOS values did not change significantly among different supplementation doses. The highest serum vitamin E levels were detected in the 2.5% group (*p* = 0.0454). The vitamin E concentrations in the other groups were not significantly different from each other. It was also observed that serum IgG concentration was higher in the 2.5% and 5% groups (*p* = 0.0251). There was no statistically significant variation between the 1% and control groups regarding their IgG concentrations. When the carrot juice was supplemented at the doses of 2.5% or 5% of drinking water, serum ALP levels increased (*p* = 0.0008), while the serum ALP levels of the hens were not different between the control and 1% supplementation groups. There were no significant changes in other biochemical parameters, including TAS, HDL, GGT, cholesterol, AST, ALT, LDL, phosphorus, total protein, glucose, or calcium concentrations among the groups.

Although the LCs were decreased in the 5% group (*p* = 0.0268), these counts were not statistically different among other supplementation groups and the control group (Table 5). Additionally, MCH levels of the groups in which drinking water was supplemented with carrot juice were higher than those of the control group (*p* = 0.036). The TLC, NC, MC, RBC, He, HC, MCV, MCHC, PLT, and MPV results did not display statistically significant differences among the groups (Table 5).

PCA values significantly increased over time in the control group, with the values measured at the end of the study being higher than those on day 15 (*p* = 0.0002). Nonetheless, no statistically significant differences were seen between day 15 and day 30 in the 1%, 2.5%, and 5% carrot juice supplementation groups (Table 6). Furthermore, no significant variations in PCA values were observed among the groups at the first or second sampling points (Table 6). The levels of MRS, SBM, and PBLS did not change significantly over time from day 15 to day 30 of the study in any of the groups (Table 6). Although no significant variations in MRS counts were observed among the groups at the first sampling point, the counts were significantly greater in the 2.5% supplementation group compared to the 1% group at the end of the study (*p* = 0.037). SBM levels measured on day 15 of the study were not significantly different among the groups. However, at the second time point of sampling, the SBM counts in the 5% supplementation group were significantly greater than those that were observed in the control group (*p* = 0.027). The PBLS and *C. perfringens* counts were not significantly influenced by carrot juice supplementation at the first or second fecal sampling time (Table 6). In the control group, *C. perfringens* counts were lower on the 30th day of the study in comparison to the 15th day. No significant differences were seen in PBLS and *C. perfringens* counts over time in any group except for the control group. *C. perfringens* counts were lower at the end of the study in comparison to the 15th day in the control group (*p* = 0.017) (Table 6).

## 4. Discussion

In this study, the addition of carrot juice to the drinking water of laying hens increased their egg production, egg weight, and egg mass values without affecting their feed intake, water consumption, or FCR. Contrary to our results, it was reported that carrot juice supplementation reduced the water intake in broiler chickens [10]. This discrepancy can result from other environmental factors and the type of birds included in the studies. On the other hand, Sikder et al. [24] stated that while the addition of dried carrot meal to laying hen diets did not have an impact on egg production, egg weight, or FCR, it reduced the amount of feed intake. De Souza et al. [25], similarly, found no significant impact of carrot meal addition on performance parameters, including egg mass, egg production, feed intake, and FCR. The main difference between these studies and this study was the form of carrot usage. It should be noted that the non-starch polysaccharide and lignin contents of carrot are higher than those in a standard laying hen diet prepared using NRC recommendations [7]. Therefore, one of the potential reasons for the absence of a significant effect of carrot meal addition on performance parameters in previous studies was that replacing any ingredient found in a standard diet with carrot meal could restrict the intake of some nutrients, such as amino acids. For this reason, carrot juice addition can have a positive effect on performance parameters, including egg production, egg mass, and egg weight, because it does not affect the intake of other nutritional constituents.

Vitamin E concentrations in the blood could be another factor affecting performance parameters. Zhao et al. [26] showed that vitamin E supplementation increased egg production and egg mass. Similarly, Sharma et al. [27] found that vitamin E supplementation positively affected egg production. In this study, although there was no statistically significant increase in all groups, carrot juice supplementation raised the serum vitamin E levels of the hens. So, the increased serum vitamin E levels may have led to an increase in egg production. In contrast, Yang et al. [28] stated that vitamin E supplementation did not influence the productivity of laying hens. Because egg production increased in all groups even if serum vitamin E levels remained unchanged, the effects of vitamin E on egg production need to be investigated further.

It is known that carrots accumulate high amounts of carotenoid pigments, which are precursors of vitamin A [29]. In a meta-analysis, it was shown that the dietary supplementation of natural carotenoid sources to laying hens improved the yolk color and Haugh unit values of their eggs [30]. Also, the inclusion of *Saccharomyces* spp., fermented carrot leaf meal in the diet can enhance the hen–day production and elevate β-carotene levels in the yolk, while concurrently reducing cholesterol content in the yolk. For this reason, the increased Haugh unit and egg yolk color values in the 2.5% and 1% groups, respectively, may have resulted from the carotene content naturally found in the carrots. In this study, there was a decrease in eggshell thickness in the 1% group. Boğa Kuru et al. [31] reported an inverse relationship between egg weight and eggshell thickness, even though shell weight increased concurrently with egg weight. Also, it should be noted that the highest values for egg production, egg weight, and egg mass were recorded in the 1% group in the current study. As egg weight increases, the deposition of shell material over a larger egg surface area may result in reduced shell thickness. Therefore, the lower eggshell thickness observed in the 1% group may be attributed, at least in part, to the increased egg weight and egg mass in this group.

In our study, carrot juice supplementation led to increases in the TOS, ALP, vitamin E, IgG, and ALP concentrations in the serum. Carotenoids reduce oxidative stress in birds through different mechanisms, including scavenging free radicals and activating antioxidant enzymes [32]. Accordingly, Gouda et al. [33] reported that the introduction of lycopene to broiler diets led to improvements in the serum concentrations of antioxidant enzymes, including catalase, superoxide dismutase, and glutathione peroxidase. Additionally, it was shown that drinking carrot juice increased TAS values and reduced lipid peroxidation and cardiovascular risk markers [11]. Contrary to the aforementioned study, we found an increase in TOS values in all carrot juice-supplemented groups in comparison to the control group. Considering previous studies, it is difficult to explain the increased TOS values; however, based on the performance parameter values presented in Table 3, it could be suggested that increased egg production may have challenged the oxidative status in the supplemented groups.

Increased vitamin E levels could be related to the tocopherol content of carrots. However, there were no linear relationships between carrot juice supplementation and serum vitamin E levels. Alpha-tocopherol absorption from the intestines is not well correlated with dietary tocopherol intake. Thus, increased intake is a restricting factor for tocopherol absorption [34]. The majority of the ALP in the serum originates from the liver and bone tissue. ALP is found on the outer surface of the cell membrane of osteoblasts and is released into the serum with increasing osteoblastic activity. So, increased osteoblastic activity leads to ALP elevation [35]. Yamaguchi & Uchiyama [36] reported that beta-cryptoxanthin, which is a carotenoid, caused increases in calcium and ALP activity in femoral bone tissue, and they suggested that some carotenoids have an anabolic effect on bone metabolism. Therefore, based on these outcomes, it could be expected that the elevated ALP activity in the experimental groups may have been caused by the increased bone turnover caused by carotenoids.

ALP found in the serum is then transported to the liver through the blood and excreted via the biliary system. Elevated serum ALP levels are an indicator of blocked extraction [35]. In our study, while there was an increase in the ALP activities of the 2.5% and 5% groups, these values were lower than those found in a previous study conducted by Osadcha et al. [37] in laying hens. Given that the production parameters were acceptable and that there was no sign of any unexpected illness, it could be inferred that the ALP levels determined in this study were within healthy limits.

In this study, carrot juice supplementation increased IgG concentrations in the 2.5% and 5% groups and reduced lymphocyte counts in the 5% group without any change in total leucocyte counts. Carrot juice has an immunomodulatory effect due to its biologically active natural compounds taking part in molecular signaling in the immune system [38]. Wang et al. [39] found that β-carotene, which is a major ingredient of carrot, enhances immunity and elevates serum IgG levels in hens. The relative abundance of lymphocytes, neutrophils, and basophils in our study was similar to that reported in a previous study, where lymphocytes were the most abundant constituent [40]. For this reason, our results confirmed that carrot juice improves the immune system and antibody secretion without impairing the physiological parameters of blood.

Besides the effects of carrot-derived products on the immune system, β-carotene, the main active constituent of carrots, affects the metabolism of other nutrients. Gautam et al. [41] found that vegetables rich in β-carotene significantly increased the bioavailability of iron and zinc found in cereals and pulses. Similarly, García-Casal [42] reported that lycopene, lutein, and zeaxanthin without vitamin A increased iron absorption in a corn- and wheat-based diet program. Considering the essential role of iron in hemoglobin synthesis [43], it could be inferred that increased MCH in the carrot juice-supplemented groups in our study could have arisen from increased iron absorption from other dietary ingredients.

In our study, in the control group, while PCA counts increased, *C. perfringens* counts decreased from the 15th day to the 30th day of the study. Nevertheless, the same trend was not observed in the carrot juice-supplemented groups. It is difficult to explain the differences in the control group observed throughout the study. However, considering the anaerobically proliferating nature of *C. perfringens* [44], the opposite relationship between total aerobic bacteria counts and perfringens counts was not a surprising change. Because of the lack of decrease in the aerobic counts in the carrot juice-supplemented groups, contrary to the control group, it could be suggested that carrot juice may show an effect that inhibits the proliferation of aerobic bacteria. Carrot juice supplementation increased the MRS counts in the 2.5% supplemented group. However, this change was not statistically significant. SBM counts were also greater in the 5% supplementation group than in the control group. It is known that the unabsorbed polyphenolic compounds and metabolites resulting from polyphenolic intake may interact with the gut microflora and modulate the microbial composition through various mechanisms of action [45]. It was demonstrated that the consumption of drinks containing flavanols had a prebiotic effect on gut microbes, and it stimulated the proliferation of some bacterial species such as *Enterococcus* and Lactobacilli [46]. These findings suggested that the phenolic compounds present in carrot juice may have prebiotic effects on the gut microbiota. One of the well-established benefits of prebiotics is their capacity to suppress the proliferation of pathogenic microorganisms, including *C. perfringens* [47]. In this study, no such effect was observed on *C. perfringens* counts. Therefore, the potential prebiotic effects of the phenolic compounds in carrot juice warrant further investigation.

Although supplementation via drinking water offers practical advantages in modern poultry production systems, certain limitations should be considered. Individual water intake may vary among hens depending on environmental and physiological conditions, potentially leading to differences in actual nutrient consumption.

## 5. Conclusions

In conclusion, carrot juice appears to be a promising natural and sustainable additive that may enhance laying hen performance while offering an alternative utilization strategy for surplus or non-marketable carrots. This study indicated that the use of carrot juice in laying hens has positive effects, potentially supporting performance and health. In current egg production systems, carrot juice can be incorporated into drinking water systems to contribute to improved productivity. Among the levels evaluated, adding 2.5% carrot juice to drinking water was found to provide the most balanced response. Future research should focus on elucidating the mechanisms underlying carrot juice-induced changes in antioxidant status, immune function, and intestinal microbiota, as well as their implications for eggshell quality.

## Figures and Tables

**Table 1:** Ingredients and nutrient composition of basal rations and the composition of the carrot juice.

Feedstuffs	% (As-Fed Basis)
Corn	56.30
Sunflower Meal (28% CP)	13.50
Soybean Meal (44% CP)	10.00
Full-Fat Soybean	9.06
Limestone	9.00
Dicalcium Phosphate	1.38
Salt	0.40
Vitamin–Mineral Premix ^a^	0.25
DL-Methionine	0.08
L-Lysine Hydrochloride	0.03
Nutrient Composition	
Dry Matter (%)	90.5
Crude Protein (%)	16.3
Metabolizable Energy (kcal/kg)	2739
Calcium (%)	3.84
Available Phosphorus (%)	0.32
Sodium (%)	0.19
Methionine + Cystine (%)	0.68
Lysine (%)	0.79
Threonine (%)	0.61
Tryptophan (%)	0.21
Analyzed Composition of Carrot Juice	
Constituent	Quantity
Glucose (g/100 g)	0.957
Fructose (g/100 g)	0.757
Saccharose (g/100 g)	3.68
Beta-carotene (mg/kg)	80.6
Total phenolic substances (mg/gallic acid), acid/100 mL)	202.7

^a^ Supplemented per kilogram of diet: Vitamin A: 12,000,000 IU; Vitamin D3: 3,000,000 IU; Vitamin E: 35,000 IU; Vitamin K3: 3500 IU; Vitamin B1: 2750 IU; Vitamin B2: 5500 IU; Nicotinamide: 30,000 IU; Ca-D-Pantothenate: 10,000 IU; Vitamin B6: 4000 IU; Vitamin B12: 15 IU; Folic acid: 1000 IU; D-Biotin: 50 IU; Choline Chloride: 150,000 IU; Manganese: 80,000 mg; Iron: 60,000 mg; Zinc: 60,000 mg; Copper: 5000 mg; Iodine: 2000 mg; Cobalt: 500 mg; Selenium: 150 mg; and Antioxidant: 15,000 mg.

**Table 2:** Methods used for microbial analysis.

Microorganism	Media	Incubation Conditions	Methods
*Lactobacillus* spp.	MRS Agar (Oxoid CM 3611) (De Man, Rogosa, Sharpe)	30 °C 24/72 h of aerobic incubation	[19]
*Enterococcus* spp.	Slanetz and Bartley Medium (SBM) (Oxoid Code: CM 377)	37 °C 24/48 h of aerobic incubation	[20]
*Salmonella* spp.	Brilliant-Green Phenol-red Lactose Sucrose Agar (PBLS) (Merck 1.07237)	35–37 °C 24/48 h of aerobic incubation	[21]
*C. perfringens*	Perfringens Agar Base (Oxoid CM 543)	37 °C 24/48 h of anaerobic incubation	[22]
Total number of aerobic bacteria	Plate Count Agar (PCA) (Oxoid CM 325)	30 °C 48/72 h of aerobic incubation	[23]

**Table 3:** Effects of carrot juice on performance parameters and egg quality in laying hens ^1^, weeks 1–4.

Item	Control	CJ 1%	CJ 2.5%	CJ 5%	SEM ^3^	Treatment	Time	Treatment x Time
Mean Daily Feed Intake (g)	117.50	125.60	120.00	123.10	3.5721	0.4056	0.3003	0.440
Mean Daily Egg Production (%)	72.28 ^b^	85.56 ^a^	81.84 ^a^	84.07 ^a^	1.9307	<0.0001	0.3426	0.052
FCR ^2^ (Kg Feed/Kg Egg Mass)	2.60	2.31	2.36	2.33	0.1216	0.0885	0.4009	0.602
Egg Weight (g)	62.63 ^b^	65.24 ^a^	63.97 ^ab^	63.64 ^ab^	0.6348	0.0452	0.1824	0.391
Egg Mass (g/Hen/Day)	45.05 ^b^	55.16 ^a^	51.96 ^a^	53.68 ^a^	1.9731	0.0037	0.9732	0.430
Water Consumption (mL/day)	370	430	410	400	59.70	0.073	0.0786	0.839
Body weight of hen (g)	1550.96 ^ab^	1573.17 ^a^	1566.20 ^a^	1499.62 ^b^	19.8405	0.0411	<0.0001	0.924
Hough Units	86.85 ^b^	86.70 ^b^	91.57 ^a^	86.06 ^b^	1.3038	0.0089	0.0013	<0.0001
Egg Yolk Color	10.04 ^b^	10.85 ^a^	10.01 ^b^	10.22 ^b^	0.1401	0.0001	0.0003	0.011
Mean Eggshell Thickness mm^−1^	41.11 ^a^	38.98 ^b^	40.68 ^a^	40.98 ^a^	0.5351	0.0213	0.387	0.058
Albumen Index	10.54 ^ab^	10.42 ^ab^	11.28 ^a^	9.90 ^b^	0.3464	0.051	0.0173	0.0006
Egg Yolk Index	47.95	43.37	42	43.25	0.0218	0.2692	0.1591	0.0397

^1^ Data are shown as least square means. The results are averages calculated for four replicate cages per ration with eight hens (n = 32). Control = no supplementation/plain water; 1% CJ = 1% carrot juice supplementation to drinking water; 2.5% CJ = 2.5% carrot juice supplementation to drinking water; and 5% CJ = 5% carrot juice supplementation to drinking water. ^2^ Feed conversion ratio = feed consumption in grams/egg mass in grams. ^3^ Standard error of means. ^a,b^ Values with multiple superscripts in the same column show significant changes (*p* < 0.05).

**Table 4:** Effects of carrot juice on serum biochemical parameters in laying hens ^1^.

Groups	Control	CJ 1%	CJ 2.5%	CJ 5%	SEM ^2^	*p*
TOS ^3^ mmol/L	21.08 ^b^	31.41 ^a^	35.69 ^a^	31.08 ^a^	2.6989	0.015
TAC ^4^ mmol/L	0.75	0.93	0.95	0.91	0.0845	0.361
VITAMIN-E mg/L	3.94 ^b^	3.92 ^b^	4.77 ^a^	4.53 ^ab^	0.2252	0.045
IgG ^5^ mg/dL	150.00 ^b^	223.75 ^ab^	373.00 ^a^	336.00 ^a^	49.703	0.025
HDL ^6^ mg/dL	13.50	13.75	14.5	15.25	0.6692	0.291
GGT ^7^ U/L	24.75	28.50	29.00	28.50	2.0349	0.451
CHO ^8^ mg/dL	96.00	69.00	122.75	131.25	18.137	0.117
AST ^9^ U/L	151.75	168.00	174.00	182.75	10.38	0.244
ALT ^10^ U/L	0.85	0.89	0.91	0.92	0.0411	0.638
LDL ^11^ mg/dL	41.75	33.00	54.50	56.50	8.8727	0.249
Phosphorus mg/dL	4.12	5.00	4.72	5.12	0.364	0.266
ALP ^12^ U/L	190.25 ^c^	220.75 ^bc^	319.25 ^a^	271.00 ^ab^	16.885	0.0008
Total Protein g/dL	5.70	6.00	5.75	5.77	0.4329	0.961
Glucose mg/dL	202.75	206.00	225.5	232.25	11.046	0.216
Calcium mg/dL	23.17	24.42	25.47	26.72	1.7827	0.559

^1^ Data are shown as least square means. The results are averages calculated for four replicate cages per ration with eight hens (n = 32). Control = no supplementation/plain water; 1% CJ = 1% carrot juice supplementation to drinking water; %2.5 CJ = %2.5 carrot juice supplementation to drinking water; and 5% CJ = 5% carrot juice supplementation to drinking water. ^2^ Standard error of means, ^3^ TOS: total oxidant status, ^4^ TAC: total antioxidant status, ^5^ IgG: immunoglobulin G, ^6^ HDL: high-density lipoprotein, ^7^ GGT: gamma glutamyl transferase, ^8^ cholesterol, ^9^ AST: aspartate aminotransferase, ^10^ ALT: alanine aminotransferase, ^11^ LDL: low-density lipoprotein, and ^12^ ALP: alkaline phosphatase. ^a,b,c^ Values with multiple superscripts in the same column show significant changes (*p* < 0.05).

**Table 5:** Effects of carrot juice on blood physiological parameters in laying hens ^1^.

Groups	Control	CJ 1%	CJ 2.5%	CJ 5%	SEM ^2^	*p*
TLC ^3^ (10^9^/L)	2.56	2.41	2.45	2.54	0.2571	0.971
LC ^4^ (10^9^/L)	1.75 ^a^	1.81 ^a^	1.72 ^ab^	1.63 ^b^	0.0411	0.026
NC ^5^ (10^9^/L)	0.80	0.68	0.78	0.76	0.0427	0.213
MC ^6^ (10^9^/L)	0.04	0.04	0.04	0.04	0.0007	0.743
RBC ^7^ (10^9^/L)	2.67	2.63	2.68	2.65	0.0498	0.886
He ^8^ (g/L)	10.47	10.63	10.52	10.54	0.2508	0.975
HC ^9^ %	34.07	33.88	34.89	34.56	0.4589	0.393
MCV ^10^ (fL)	108.28	108.42	108.19	109.01	0.5314	0.698
MCH ^11^ (pg)	29.34 ^b^	31.21 ^a^	31.03 ^a^	31.14 ^a^	0.5087	0.036
MCHC ^12^(g/L)	30.93	30.75	30.89	30.92	0.5877	0.995
PLT ^13^ (10^9^/L)	27.59	26.70	27.00	27.13	0.5075	0.659
MPV ^14^ (pg)	6.40	6.55	6.47	6.47	0.0814	0.648

^1^ Data are shown as least square means. The numbers are an average of four replicate cages per ration with eight hens (n = 32). Control = no supplementation/plain water; 1% CJ = 1% carrot juice supplementation to drinking water; 2.5% CJ = 2.5% carrot juice supplementation to drinking water; and 5% CJ = 5% carrot juice supplementation to drinking water. ^2^ Standard error of means, ^3^ TLC: total leukocyte count, ^4^ LC: lymphocyte count. ^5^ NC: neutrophil count, ^6^ MC: monocyte count, ^7^ RBC: red blood cell count, ^8^ He: hemoglobin, ^9^ HC: hematocrit, ^10^ MCV: mean corpuscular volume, ^11^ MCH: mean corpuscular hemoglobin, ^12^ MCHC: mean corpuscular hemoglobin concentration, ^13^ PLTs: platelets, and ^14^ MPV: mean platelet volume. ^a,b^ Values with multiple superscripts in the same column show significant changes (*p* < 0.05).

**Table 6:** Average bacterial populations of fecal samples in laying hens (effects of carrot juice on PCA, MRS, SBM, PBLS, and *C. perfringens*) (log10 cfu/g).

Group		Control	CJ 1%	CJ 2.5%	CJ 5%	*p*
		$\bar{x}$ $\;\text{±}\;S\bar{x}$	$\bar{x}$ $\;\text{±}\;S\bar{x}$	$\bar{x}$ $\;\text{±}\;S\bar{x}$	$\bar{x}$ $\;\text{±}\;S\bar{x}$	
PCA	1st Measurement	18.33 ± 4.77	48.50 ± 2.31	47.33 ± 17.55	80.67 ± 27.13	0.343
	2nd Measurement	102.08 ± 35.07	53.08 ± 9.89	60.67 ± 17.50	103.42 ± 25.04	0.368
	*p*	0.002	0.375	0.181	0.264	
MRS	1st Measurement	109.83 ± 34.75	27.33 ± 13.10	115.83 ± 41.53	69.42 ± 29.12	0.117
	2nd Measurement	51.75 ± 24.87 ^ab^	10.58 ± 5.02 ^b^	83.08 ± 30.42 ^a^	34.25 ± 9.29 ^ab^	0.037
	*p*	0.294	0.496	0.963	0.846	
SBM	1st Measurement	13.42 ± 6.17	33.17 ± 14.31	47.42 ± 18.29	57.50 ± 20.34	0.209
	2nd Measurement	34.58 ± 4.32 ^b^	34.83 ± 9.80 ^ab^	28.25 ± 12.16 ^ab^	120.92 ± 33.38 ^a^	0.027
	*p*	0.956	0.132	0.364	0.148	
PBLS	1st Measurement	0.083 ± 0.001	0.500 ± 0.001	0.500 ± 0.359	2.250 ± 1.754	0.369
	2nd Measurement	0.083 ± 0.002	0.167 ± 0.112	n.d.	1.333 ± 0.932	0.164
	*p*	1.000	0.522	0.177	0.649	
*C. perfringens*	1st Measurement	11.75 ± 4.23	6.00 ± 2.42	4.08 ± 1.58	4.67 ± 1.63	0.179
	2nd Measurement	0.75 ± 0.66	3.92 ± 1.76	6.92 ± 1.06	13.08 ± 3.85	0.070
	*p*	0.017	0.493	0.598	0.056	

PCA: Total aerobic plate count; MRS: *Lactobacillus* spp.; SBM: *Enterococcus* spp.; PBLS: *Salmonella* spp.; n.d.: not detected. $\bar{x}$: Mean; S$\bar{x}$: standard error of the mean. ^a,b^ Values with multiple superscripts in the same column show significant changes (*p* < 0.05).

## Data Availability

Data is contained within the article.
